# A 3D-Printed Multichannel Viscometer for High-Throughput Analysis of Frying Oil Quality

**DOI:** 10.3390/s18051625

**Published:** 2018-05-19

**Authors:** Sein Oh, Byeongyeon Kim, Sungyoung Choi

**Affiliations:** Department of Biomedical Engineering, Kyung Hee University, 1732 Deogyeong-daero, Giheung-gu, Yongin-si, Gyeonggi-do 17104, Korea; hottaek_90@khu.ac.kr (S.O.); kby921022@khu.ac.kr (B.K.)

**Keywords:** viscometer, multiplexed analysis, quality control, frying oil quality, microfluidics, 3D printing

## Abstract

Viscosity as a sensitive measure of material changes is a potential quality-control parameter for simple and rapid assessment of frying oil quality. However, conventional viscometers require improvements in throughput, portability, cost-effectiveness and usability to be widely adopted for quality-control applications. Here we present a 3D-printed multichannel viscometer for simple, inexpensive and multiplexed viscosity measurement. The multichannel viscometer enables both parallel actuation of multiple fluid flows by pressing the plunger of the viscometer by hand and direct measurement of their relative volumes dispensed with naked eye. Thus, the unknown viscosities of test fluids can be simultaneously determined by the volume ratios between a reference fluid of known viscosity and the test fluids of unknown viscosity. With a 4-plex version of the multichannel viscometer, we demonstrated that the viscometer is effective for rapid examination of the degradation of a vegetable oil during deep frying of potato strips and the recovery of used frying oil after treatment with an adsorbent agent to remove frying by-products. The measurement results obtained by the multichannel viscometer were highly correlated with those obtained using a commercial oil tester. We also demonstrated the multiplexing capability of the viscometer, fabricating a 10-plex version of the viscometer and measuring the viscosities of ten test liquids at the same time. Collectively, these results indicate that the 3D-printed multichannel viscometer represents a valuable tool for high-throughput examination of frying oil quality in resource-limited settings.

## 1. Introduction

Examining the degradation of frying oil is essential to produce safe and high-quality fried foods because their safety and taste depend on the frying oil quality [1,2]. Different analytical techniques have been used to determine frying oil deterioration by chemically or physically measuring quality parameters such as total polar materials (TPM). These methods are based on chromatographic separation [3], near-infrared spectroscopy [4,5], and changes in electrical properties [6,7]. Although these techniques have been proven to be effective for monitoring the degradation degree of used frying oil, each of these techniques has its intrinsic limitations. Chromatographic and spectroscopic approaches typically require time-consuming and laborious sample preparation, bench-top laboratory equipment, and trained personnel for instrumental operation and maintenance, thus limiting their practical utility in field-based settings. Electrical approaches are widely used in the food industry because the electrical properties of frying oil can be easily and quickly measured using portable electronic devices. However, additional ingredients such as salts, water, and minerals can adversely affect electrical measurement results [8]. In addition, the use of a single sensor probe and its cleaning requirement before every measurement inherently limit the throughput capability for frying oil testing. Alternatively, viscosity as a sensitive measure of material changes is a potential parameter for simple and rapid evaluation of frying oil quality based on a strong correlation between viscosity changes and TPM [8,9]. However, conventional viscometers typically rely on bulk and expensive equipment [10,11], thereby making them unsuitable for field-deployable, cost-effective and high-throughput analysis of frying oil quality.

Microfluidic viscometers can address the challenges of conventional viscometers by taking advantages of microfluidics such as small device footprint, ease of use, and low sample consumption. They can be categorized by their geometry: a simple capillary channel [12,13], a microfluidic comparator [14,15,16,17,18], a droplet generator [19,20], a flow focuser [21], and a pressure sensor-integrated microchannel [22]. Viscometers that are based on a simple capillary channel utilize the dependence of the resulting fluid velocity of a test liquid on its viscosity under a known capillary pressure [12,13]. Microfluidic comparators employ the co-flowing laminar streams of two different liquids: one with known viscosity and the other with unknown viscosity [14,15,16,17,18]. Since the fluid streams in the comparators depend on their viscosity ratio, the unknown viscosity can be determined by comparing their relative flow rate or the equilibrium position of the fluid interface. Droplet-based viscometers have been developed by exploiting the fact that the size of water-in-oil droplets generated through a micro-nozzle is highly affected by aqueous-phase and oil-phase viscosities [19,20]. A microfluidic flow focuser has been used to measure viscosity by monitoring the diffusion of tracer particles under a laminar flow condition [21]. A pressure sensor-integrated microchannel has been developed for accurate measurement of the pressure profile along a microchannel at a given flow rate and subsequent viscosity calculation based on the Hagen-Poiseuille law [22]. Although these methods enable simple viscosity measurement, they typically rely on either accurate generation of a pressure drop and a volumetric flow rate through a microfluidic device or delicate control of multiple fluid streams, inevitably requiring bulky and expensive pumping equipment for device operation and cumbersome microscopic flow observation for viscosity measurement. In addition, most of them lack the ability to perform multiplexed viscosity measurement. A microfluidic viscometer integrated with multiple comparators has been demonstrated with the capability of simultaneously measuring the viscosities of multiple fluids [23]. However, its further throughput improvement seems to be limited by its complexities in device operation that are significantly increased with the number of fluids to be tested. Thus, there still remains an unmet need for the development of a low-cost, accurate, multiplexed and field-deployable viscometer to be used for the routine quality testing of frying oil.

**Figure 1:**
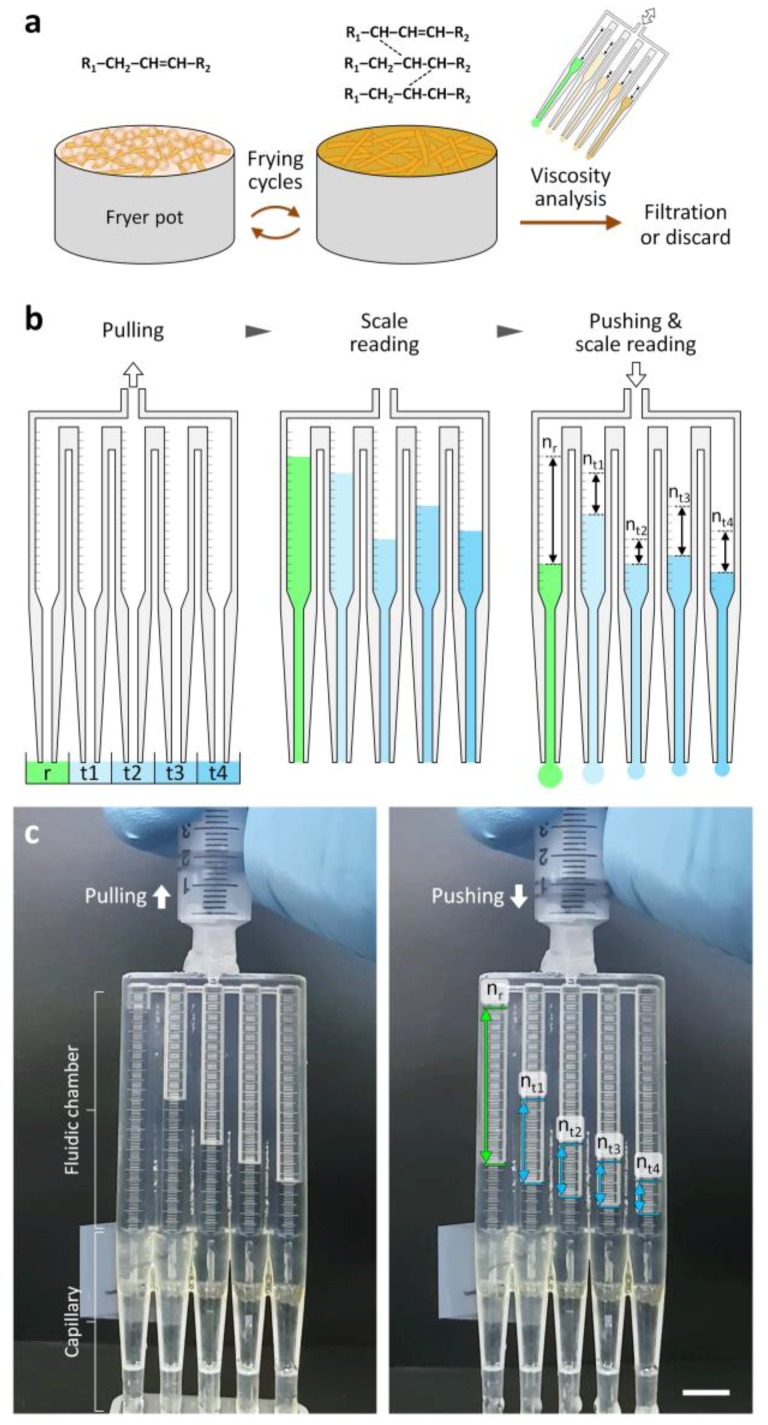
3D-printed multichannel viscometer for simple and rapid analysis of frying oil quality. (**a**) Dimers, polymers and cyclic compounds form during deep frying, resulting in an increase in frying oil viscosity that can be simply assessed using the multichannel viscometer. (**b**) Schematics showing the viscosity measurement process. The unknown viscosities of the test fluid samples can be determined by the ratios of dispensed volumes from the reference and test channels. (**c**) Sequence of photographs showing that the dispensed volume from each channel depends on liquid viscosity. For the operation of the multichannel viscometer, the syringe was pushed until the liquid meniscus descends more than ten graduations in a fluidic chamber of the viscometer. Four different liquids can be simultaneously tested. Glycerol-water mixtures at different weight ratios were aspirated and dispensed. Their viscosity values were 20.3, 38.2, 60.4, 78.0, and 98.5 cP in the order from left to right. Subscripts *r* and *t* represent reference and test, respectively. Scale bar, 1 cm.

To address these challenges, we have developed a 3D-printed multichannel viscometer for rapid and parallel viscosity measurement of multiple fluids by intuitive operation and measurement protocols. The 3D-printed capillary circuit was recently developed by proving its viscosity measurement principle for the first time and measuring the viscosity of a test fluid at a certain time [24]. When applying this measurement method to field-based applications, parallel measurement units need to be integrated to achieve sufficient measurement throughput and thus allow rapid viscosity measurement. We thus engineered the 3D-printed multichannel viscometer by incorporating five or eleven channels in a device. The new viscometer design provides simple but accurate control (i.e., aspiration and dispensing) of multiple fluids by sequentially generating common negative and positive pressure with a commercial syringe as well as precise comparison of their viscosities by reading the descending difference of each fluid column with naked eye (Figure 1). Using a 10-plex version of the multichannel viscometer, we demonstrated significant improvement in viscosity measurement throughput by measuring up to ten test liquids at the same time. With a 4-plex version of the multichannel viscometer, we investigated the correlation between viscosity changes and TPM of used frying oil. Moreover, the recovery of used frying oil by treatment with an adsorbent agent to remove frying by-products was successfully evaluated, demonstrating the effectiveness of the multichannel viscometer as a quality-control tool for frying oil.

## 2. Materials and Methods 

### 2.1. Design and Fabrication of the Multichannel Viscometer

**Figure 2:**
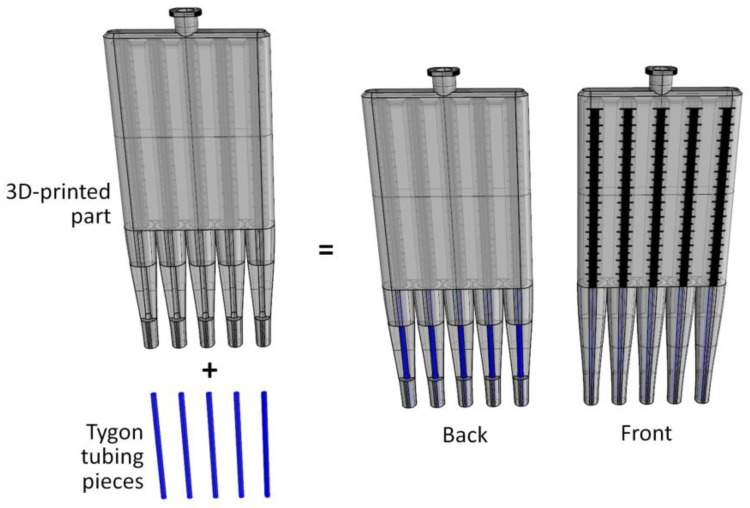
Fabrication of a 4-plex version of the multichannel viscometer. Tygon tubing pieces were assembled with a 3D-printed chamber part to form a uniform and high-resistance capillary in each channel.

The multichannel viscometer was fabricated by assembling a 3D-printed fluidic chamber part and 3.6 cm-long Tygon tubing pieces (0.508 mm in inner diameter) (Figure 2). The 3D-printed part was fabricated using a 3D printer (DWS systems, Thiene, Italy), containing five or eleven fluidic chambers connected to a common pneumatic port with a female Luer-Lock connection fitting. A BD syringe (BD Biosciences, San Jose, CA, USA) with a male Luer-Lock fitting was connected with the common pneumatic port, thereby making their leak-free interconnection. Each fluidic chamber was designed to have graduations along the chamber (Figure 1c), thereby enabling naked-eye readout of volumetric signals. The cross-section of the fluidic chamber is elliptical and its cross-sectional area is 13.1 mm^2^. Tygon tubing with an inner diameter of 0.508 mm (Cole-Parmer, Vernon Hills, IL, USA) was cut into pieces after marking the length (3.6 cm) measured with a ruler. Each tubing piece was attached at the end of each fluidic chamber using a liquid adhesive, functioning as a capillary of high hydrodynamic resistance (Figure 2). The 3D-printed housings for the multichannel viscometers were designed to have long semicircular shaped grooves that were complementary to the cut tubing pieces. A cyanoacrylate-based liquid glue (3M Corp., Saint Paul, MN, USA) was used for completely filling the gaps between the tubing pieces and 3D-printed housings. The multichannel viscometers were reused up to 5 times without performance degradation. Detergent cleaning is required to reuse the viscometers. Visual scale measurements for viscosity determination were performed using two series of photographs taken during each experiment. The distance between neighboring graduations is 0.4 mm. The discretization error of the multichannel viscometer can be defined as the viscosity value corresponding to the unit scale. When applying the following experimental results, the discretization error is estimated at 0.28 cP/scale for measuring the viscosity of 20.3 cP solution, small enough not to affect the viscosity measurement using the multichannel viscometer. A 3D printing technology was chosen for the fabrication of the multichannel viscometers. However, the capillary parts of the viscometers were made by assembling the cut tubing pieces into 3D-printed housings due to the resolution limitation of the 3D printer used in this study. For the mass production of the viscometers, precision manufacturing technologies (i.e., injection molding and hot-embossing) can be used to reduce fabrication cost and improve fabrication resolution.

### 2.2. Sample Preparation

Glycerol-water mixtures (Sigma-Aldrich, St. Louis, MO, USA) at different concentrations were prepared for characterization of the multichannel viscometer. They were also used as reference fluids. Dietary oils including corn, olive and coconut oils were purchased from CJ CheilJedang (Seoul, Korea) and KoreaSimilac (Hanam, Korea). The viscosities of oil samples and reference aqueous fluids were measured using a cone-plate viscometer (Brookfield AMETEK, Inc., Middleborough, MA, USA) for comparison with the viscosity results measured using the multichannel viscometer. Used frying oil samples were prepared by frying potato strips in corn oil (1.2 L). Fresh potato strips (150 g) were fried and 20 mL of frying oil samples were collected every frying cycle (30 min). Eight frying cycles were performed. Samples at the zero, second, fourth, sixth and eighth cycles were further analyzed for TPM and viscosity. Before oil analysis, all oil samples were filtered to remove food debris. A silicon dioxide powder was purchased from Birdie (Seoul, Korea) and used as an adsorbent to remove frying by-products (i.e., TPM) generated by the deep-frying process and improve the quality of the frying oil sample collected at the eighth frying cycle. The powder (18.6 g) was dissolved in 62.0 g of the frying oil sample at the eighth cycle and mixed well by magnetic stirring at 25 °C. After 30 min of adsorbent treatment, the recovered oil samples were then centrifuged at 4000 rpm for 15 min to remove solid debris. The TPM values of oil samples were measured using a VITO oil tester (Vito Filtration Systems, Richmond Hill, ON, Canada) an electronic device for TPM measurement. All experiments except coconut oil experiments were performed at room temperature (20–21 °C). The viscosity of coconut oil was measured at 25 °C due to its high melting point.

### 2.3. Computational Fluid Dynamics Simulation

**Figure 3:**
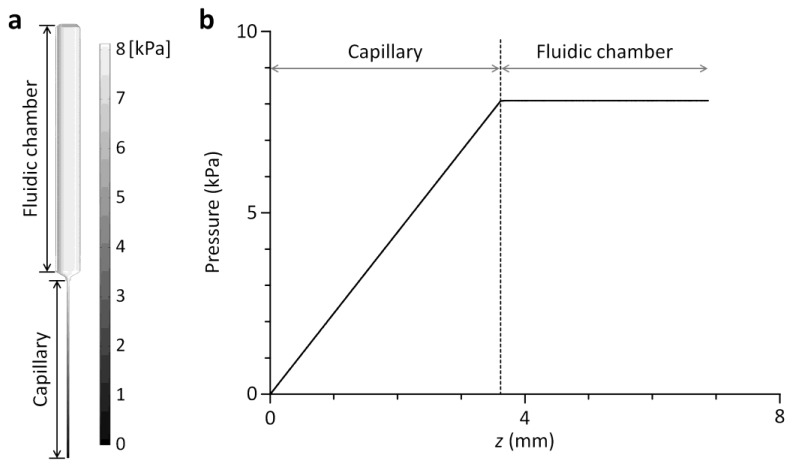
Computational fluid dynamics simulation. (**a**) Surface plot showing the pressure profile along a channel of the multichannel viscometer at the flow rate of 964.5 µL/min. (**b**) The pressure drop along the chamber is negligible compared to the drop along the capillary.

A pressure-drop profile along a fluidic channel of the viscometer was numerically analyzed using Comsol Multiphysics (Comsol, Burlington, MA, USA) (Figure 3). A three-dimensional finite element model was created in the same dimension as the channel. The capillary was 0.508 mm (diameter) × 3.6 cm (length) and the fluidic chamber was 4 mm (equivalent diameter) × 5 cm (length). The model was solved with incompressible Navier-Stokes equation, setting the flow rate to 964.5 µL/min, the viscosity to 20.3 cP and a fixed outlet pressure condition (atmospheric pressure). All surfaces except the inlet and outlet were set to no-slip boundary conditions. The numerical simulation was performed to verify the design principle of the multichannel viscometer. The hydrodynamic resistance of the chamber part should be low enough to be negligible compared to the capillary resistance. The flow rate of 964.5 µL/min for the numerical simulation was an experimental condition for measuring the viscosity (20.3 cP) of a glycerol-water mixture. The flow rate was estimated by dividing the dispensed volume during viscometer operation by the elapsed time.

## 3. Results and Discussion

### 3.1. Design Principle of the Multichannel Viscometer

Throughput improvement in viscosity measurement is required to test a large number of frying oil samples produced in the food industry. However, they are still limited by the difficulty of simultaneously operating and measuring multiple fluids with high precision. To overcome such limitation, we integrated multiple fluidic channels (five or eleven channels) for viscosity measurement into the 3D-printed multichannel viscometer (Figure 1 and Video S1), adopting the design feature of the 3D-printed capillary circuits for measurement of single test-fluid viscosity [24]. Specifically, each fluidic channel was composed of a fluidic chamber with graduated scales for simple volume measurement with naked eye and a capillary with high hydrodynamic resistance to induce dominant viscous friction. The capillary radius (*r*_c_) was set at 0.254 mm to satisfy the design rule of the 3D-printed capillary circuits for accurate viscosity measurement [24]:

(1) $$\frac{L_{c}}{r_{c}^{4}}>410\frac{L_{f}}{r_{f}^{4}}$$

where *L*_c_ is the capillary length, *L*_f_ is the length of a fluid filling the fluidic chamber, and *r*_f_ is the radius of the fluidic chamber. At *L*_c_ = 36.5 mm, *L*_f_ = 30.8 mm, *r*_c_ = 0.254 mm, and *r*_f_ = 2.0 mm, the pressure drop across the fluidic chamber was negligible so that a fluid flow through each fluidic channel could be determined solely by the capillary (Figure 3). In the parallel configuration of multiple fluidic channels (Figure 1), the pressure drop across each capillary could be defined as:

(2) $$\Delta P=\frac{8\mu_{r}L_{c}Q_{r}}{\pi r_{c}^{4}}=\frac{8\mu_{t1}L_{c}Q_{t1}}{\pi r_{c}^{4}}=\cdots =\frac{8\mu_{t4}L_{c}Q_{t4}}{\pi r_{c}^{4}}$$

where ∆*P* is the pressure drop through each capillary, *µ* is the liquid viscosity, *Q* is the volumetric flow rate, and subscripts *r* and *t* denote a reference fluid of known viscosity and a test fluid of unknown viscosity, respectively. Thus, Equation (2) can be rewritten to simultaneously determine the viscosities of multiple test fluids as follows:

(3) $$\mu_{t1}=\left(\frac{Q_{r}}{Q_{t1}}\right)\mu_{r}=\left(\frac{\pi n_{r}r_{f}^{2}/t}{\pi n_{t1}r_{f}^{2}/t}\right)\mu_{r}=\frac{n_{r}}{n_{t1}}\mu_{r},\cdots ,\mu_{t4}=\frac{n_{r}}{n_{t4}}\mu_{r}$$

where *n* is the length difference between the start and end positions of the meniscus during liquid dispensing and *t* is the time elapsed during liquid dispensing. Such simple measurement of reading scales with naked eye and calculating the ratios of dispensed volumes of a reference fluid and test fluids enable inexpensive, accurate, multiplexed and field-deployable viscosity measurement.

### 3.2. Characterization of the Multichannel Viscometer

**Figure 4:**
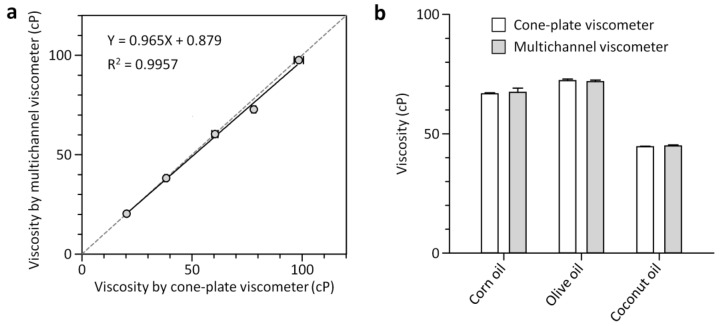
Characterization of the 4-plex multichannel viscometer. (**a**) Comparison of the multichannel and cone-plate viscometer for viscosity measurement of glycerol-water mixtures. Solid and dotted lines represent a linear regression fit and a unity slope, respectively. (**b**) Viscosity measurement results for various oils, measured with the multichannel and cone-plate viscometer. Error bars: s.d. (*n* = 3).

We first tested the capability of the 4-plex multichannel viscometer to simultaneously measure viscosities of multiple test fluids ranging from 20.3 to 98.5 cP (Figure 1 and Figure 4). Glycerol-water mixtures of different weight ratios (68 to 84 wt.%) were prepared and their viscosities were determined using the cone-plate viscometer to characterize the multichannel viscometer. The simple operation of aspirating and dispensing a reference fluid and test fluids through the multichannel viscometer enabled accurate and multiplexed viscosity determination of glycerol solutions. A glycerol solution with viscosity of 60.4 ± 1.4 cP was used as a reference fluid. The viscosity of the reference fluid was set to be similar to that of the test fluid. Since an unknown viscosity is determined by the ratio of *n*_ref_ to *n*_test_, the measurement accuracy might depend on the number of scales counted. The ratio was more than 0.33 and less than 2.1 for all the experiments that resulted in precise measurements with the coefficient of variation (CV) of 2.2% on average. We tested all the multichannel viscometers with deionized water before every experiment and confirmed that there were no inter-channel variations for viscosity measurement. As shown in Figure 4a, the viscosity results obtained using the multichannel viscometer and the cone-plate viscometer were closely matched across the viscosity range from 20.3 to 98.5 cP, with a linear-regression coefficient of 0.965 and an *R*^2^ value of 0.9957. The measurement range of the multichannel viscometer can be adjusted by changing the reference viscosity. The range can be larger than one fourth times of the reference viscosity and smaller than 4 times, as previously described [24]. Parallel pneumatic operation of separate liquid columns in the multichannel viscometer also allowed for rapid and multiplexed measurement of oil viscosity, addressing the challenge associated with interfacial instabilities that might arise between immiscible fluids (i.e., reference aqueous and test oil fluids) and make it difficult to directly compare the immiscible fluids flowing next to each other. As can be seen in Figure 4b, the viscosity values of three different oils determined by the multichannel viscometer were in good agreement with those measured using the cone-plate viscometer. We found that coconut oil had the lowest viscosity of 44.6 cP among all oils. This is due to the dependence of viscosity on the chemical structure (i.e., length and hydrophobicity) of liquid molecules and their interactions. Coconut oil consists of mostly lauric acid, a 12-carbon chain fatty acid. Other oils are composed of longer chains of carbon atoms (about 16 atoms) [25,26]. At longer chain lengths, intermolecular interactions such as van der Waals forces become stronger that can result in an increase in viscosity [25]. We also noted that the multichannel viscometer took 3 min to run and analyze four test samples while the cone-plate viscometer took longer time (12 min) due to the use of a single rotating cone and its cleaning requirement before every measurement. These results demonstrate the effectiveness of the multichannel viscometer for easy and rapid analysis of multiple fluids. Considering that viscosity is a potential quality-control parameter for examining frying oil quality and a large number of samples are produced in the food industry that needs to be tested, the simplicity and multiplexing feature of the multichannel viscometer can facilitate its industrial translation.

### 3.3. Quantitative Assessment of Frying Oil Degradation

**Figure 5:**
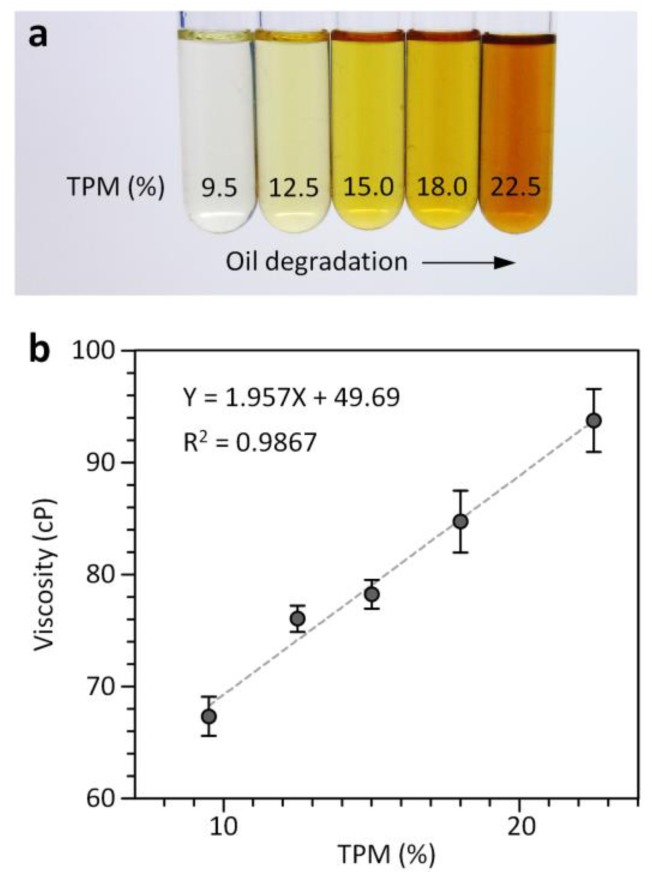
Analysis of frying oil quality using the 4-plex multichannel viscometer. (**a**) The degradation of corn oil by repeated frying is accompanied with an increase in TPM as well as color change. (**b**) Correlation between TPM values measured by the oil tester and viscosity values determined by the multichannel viscometer. Error bars: s.d. (*n* = 3).

During deep frying, a number of chemical reactions (i.e., oxidation, hydrolysis, isomerization, and polymerization) can occur between frying oil, atmospheric oxygen and water contained in foods. These can create unhealthy by-products such as polar compounds, free fatty acids and polymeric materials (dimers, polymers and cyclic compounds), thereby degrading frying oil quality [25,26,27,28]. Thus, it has been recommended that frying oil should be discarded before TPM reach their general limits of 24% [8,29]. Viscometers can allow rapid evaluation of frying oil quality based on a strong correlation between viscosity changes and TPM [8,9]. However, they are incapable of measuring oil viscosity in a cost-effective, high-throughput and user-friendly manner. To address the challenge, we applied the 4-plex multichannel viscometer to simultaneously evaluate the degradation degrees of multiple frying oil samples collected at different frying cycles. Corn oil samples were obtained from the frying process of potato strips and TPM values of these samples were measured using the cooking oil tester (Figure 5a). A glycerol-water mixture with a viscosity of 39.9 ± 0.3 cP was used as an aqueous reference fluid for viscosity measurement of the oil samples. As shown in Figure 5b, the viscosity values of the corn oil samples measured using the multichannel viscometer are linearly correlated with their TPM values measured using the cooking oil tester, in agreement with previous results obtained using a droplet-based viscometer [19].

**Figure 6:**
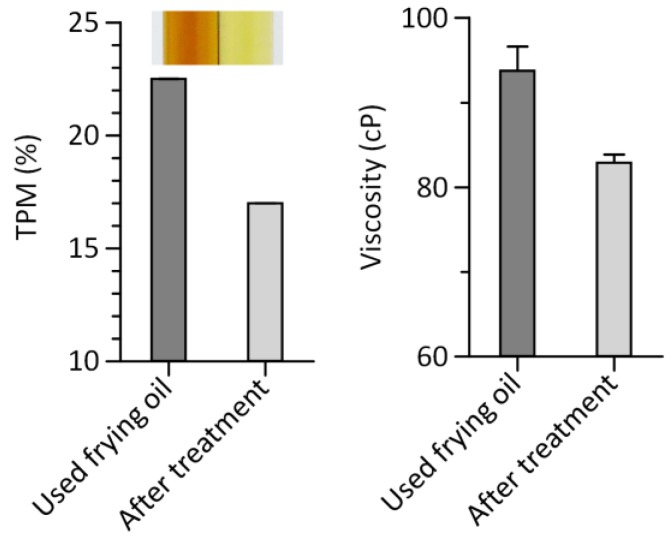
Regeneration of used frying oil after treating the oil with a silicon dioxide powder as an adsorbent in terms of TPM (**left**) and viscosity (**right**). The inset shows the color change of used frying oil after the adsorbent treatment. Error bars: s.d. (*n* = 3).

The recovery of used frying oils can extend the frying life of oil, improving the quality of fried foods. Oil recovery can be accompanied by changes in color (becoming lighter) (Figure 6). Such alteration can be used as an indicator of oil quality. However, it may not be appropriate for its general use due to diversity of oil color [29]. To address the above need, we tested whether the multichannel viscometer was capable of evaluating the effect of adsorbent treatment on oil quality (Figure 6).

A silicon dioxide powder was chosen as an adsorbent to remove frying by-products to improve the quality of used frying oil by eliminating undesirable substances such as TPM [30]. After the adsorbent treatment, we observed an improvement in oil quality in terms of TPM as well as a decrease in viscosity (Figure 6), supporting the effect of the adsorbent on improving oil quality and the capability of the multichannel viscometer for evaluating oil quality. These results demonstrate that the multichannel viscometer is an effective means to monitor frying oil quality in a multiplexed manner. It can be used as a potential substitute of conventional oil testers. Quantifying the degradation of liquid products by reuse or during storage is an important tool to use the products in the best condition and determine their shelf life. For instance, lubricating oils such as engine oils should be regularly replaced to minimize wear on the moving parts of an engine [31]. Formulating fluid foods at a desired viscosity is important for patients with dysphagia or swallowing disorders [32]. We anticipate that the multichannel viscometer is useful for these applications and can be easily extended to other applications that require viscosity measurement.

### 3.4. 10-Plex Measurement of Oil Viscosity

**Figure 7:**
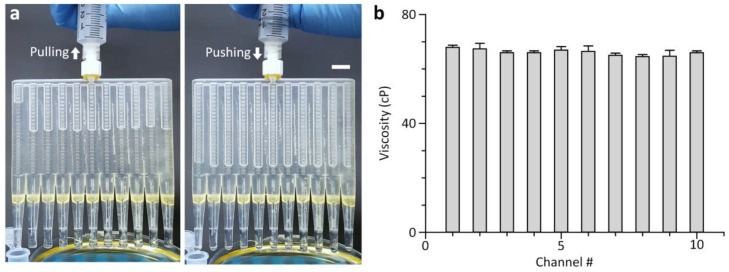
Viscosity measurement of corn oil using the 10-plex multichannel viscometer with ten test channels and a reference channel. (**a**) Sequence of photographs showing 10-plexed viscosity measurement. Of the total of eleven channels, the left-most channel was used for a reference fluid while the remaining channels were used for test fluids. Scale bar, 1 cm. (**b**) Ten concurrent viscosity measurements of corn oil without significant inter-channel variations. Error bars: s.d. (*n* = 3).

The ability to compare multiple fluid flows without inter-channel interference enables highly scalable viscosity measurement. Using this functionality, we fabricated a 10-plex version of the multichannel viscometer incorporating ten test channels and a single reference channel (Figure 7 and Video S2). It enabled simultaneous viscosity measurement of ten test fluids. A glycerol-water mixture with a viscosity of 39.9 ± 0.3 cP was used as an aqueous reference fluid for high-throughput viscosity measurement. Figure 7 shows that the multichannel viscometer is capable of measuring oil viscosity with high accuracy and throughput. The coefficient of variation for the average viscosity values obtained from each fluidic channel of the multichannel viscometer was 1.7%, indicating the consistency of the 10-plex viscosity measurement without significant inter-channel variations. The viscosity values of corn oil determined by the multichannel viscometer (66.1 ± 1.1 cP) were also in good agreement with those measured by the cone-plate viscometer (66.7 ± 0.5 cP). Further enhancement in measurement throughput can be easily achieved due to the scalability of the multichannel viscometer.

## 4. Conclusions

In summary, we demonstrated the 3D-printed multichannel viscometer for high-throughput viscosity measurement, by comparing multiple fluid flows without inter-channel interference. The proposed platform enables inexpensive, accurate, high-throughput and field-deployable measurement of oil viscosity. With these advantages, we demonstrated that changes in oil viscosity during deep frying and after adsorbent treatment were highly correlated with changes in TPM. These results open up the potential of the multichannel viscometer for routine use in oil quality assessment. We anticipate that the multichannel viscometer is useful for a wide range of industrial applications, ranging from viscosity analysis of food products to degradation assessment of various oils.

## Supplementary Materials

The following are available online at http://www.mdpi.com/1424-8220/18/5/1625/s1, Video S1: Hand-powered operation of the 4-plex multichannel viscometer, Video S2: Hand-powered operation of the 10-plex multichannel viscometer.
